# *In vitro* study on the effect of peucedanol on the activity of cytochrome P450 enzymes

**DOI:** 10.1080/13880209.2021.1944223

**Published:** 2021-07-10

**Authors:** Cun Zhang, Yongwei Li, Changlong Yin, Jie Zheng, Guozhi Liu

**Affiliations:** Department of Neonatology, Yidu Central Hospital of Weifang, Weifang, China

**Keywords:** CYP1A2, CYP2D6, CYP3A4, drug-drug interaction

## Abstract

**Context:**

Peucedanol is a major extract of *Peucedanum japonicum* Thunb. (Apiaceae) roots, which is a commonly used herb in paediatrics. Its interaction with cytochrome P450 enzymes (CYP450s) would lead to adverse effects or even failure of therapy.

**Objective:**

The interaction between peucedanol and CYP450s was investigated.

**Materials and methods:**

Peucedanol (0, 2.5, 5, 10, 25, 50, and 100 μM) was incubated with eight human liver CYP isoforms (CYP1A2, 2A6, 3A4, 2C8, 2C9, 2C19, 2D6, and 2E1), in pooled human liver microsomes (HLMs) for 30 min with specific inhibitors as positive controls and untreated HLMs as negative controls. The enzyme kinetics and time-dependent study (0, 5, 10, 15, and 30 min) were performed to obtain corresponding parameters *in vitro*.

**Results:**

Peucedanol significantly inhibited the activity of CYP1A2, 2D6, and 3A4 in a dose-dependent manner with IC_50_ values of 6.03, 13.57, and 7.58 μM, respectively. Peucedanol served as a non-competitive inhibitor of CYP3A4 with a *K_i_* value of 4.07 μM and a competitive inhibitor of CYP1A2 and 2D6 with a *K_i_* values of 3.39 and 6.77 μM, respectively. Moreover, the inhibition of CYP3A4 was time-dependent with the *K_i_*/*K_inact_* value of 5.44/0.046 min/μM.

**Discussion and conclusions:**

*In vitro* inhibitory effect of peucedanol on the activity of CYP1A2, 2A6, and 3A4 was reported in this study. As these CYPs are involved in the metabolism of various drugs, these results implied potential drug-drug interactions between peucedanol and drugs metabolized by CYP1A2, 2D6, and 3A4, which needs further *in vivo* validation.

## Introduction

Herbal medicine is one of the most common treatment methods in paediatrics in traditional Chinese medicine, which plays a crucial role in the Chinese healthcare system and has been used as alternative medicine worldwide. Unlike Western therapeutics, herbal medicines are complex mixtures, and these complex mixtures may interact with each other (Ge 2019). Besides the known pharmacological activities of *Peucedanum japonicum* Thunb. (Apiaceae), including antiallergic effects, cardiopulmonary protection, and antitumor activity (Ren et al. 2013), *Peucedanum japonicum* roots are often prescribed against cough, colds, headaches, and antifebrile in paediatrics (Sarkhail 2014). Peucedanol mainly exists in the root of *Peucedanum japonicum*, which is a commonly used herb in paediatrics (Hong and Kim 2017). The effect of peucedanol on the activity of cytochrome P450 (CYP450) enzymes is critical to guide the clinical prescription of peucedanol.

CYP450 enzymes are membrane-bound hemoproteins, which are essential for the metabolism of medications, detoxification of xenobiotics, and cellular metabolism (Guengerich et al. 2016). Induction or inhibition of CYP450 enzymes is a major mechanism that results in drug-drug interaction (Lynch and Price 2007). CYP450 enzymes can be transcriptionally activated or inhibited by various xenobiotics and endogenous substrates. Previously, a number of herbs have been reported to exert inhibitory effects on the activity of CYP450 enzymes, such as bergenin, pulvinic acid, and natural furanocoumarins (Guo et al. 2000; Huang et al. 2009; Dong et al. 2018). Drug-drug interactions induced by changes of CYP450 activity were also assessed in many studies (Yamamoto et al. 2017; Giri et al. 2018; Nishihara et al. 2019). Therefore, it is necessary to investigate the interaction between peucedanol and CYP450 enzymes to state the effect of peucedanol on the activity of CYPs, which can provide more guidance for the clinical application and prescription of peucedanol.

Peucedanol was incubated with eight major CYP isoforms (CYP1A2, 2A6, 3A4, 2C8, 2C9, 2C19, 2D6, and 2E1) in pooled human liver microsomes (HLMs). The effect of peucedanol on the activity of CYPs was assessed by a series of probe substrates and reactions. Moreover, enzyme kinetic studies and time-dependent studies were performed to obtain the potential mode of peucedanol on CYP enzymes.

## Materials and methods

### Chemicals

**Figure 1.**
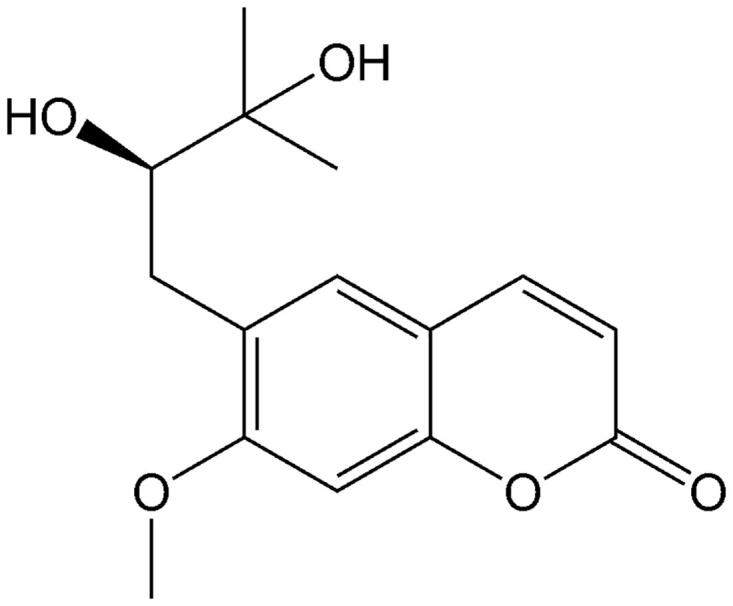
The chemical structure of peucedanol.

Peucedanol (≥98%, Figure 1) and testosterone (≥98%) were purchased from the National Institute for the Control of Pharmaceutical and Biological Products (Beijing, China). d-Glucose-6-phosphate, glucose-6-phosphate dehydrogenase, corticosterone (≥98%), NADP^+^, phenacetin (≥98%), acetaminophen (≥98%), 4-hydroxymephenytoin (≥98%), 7-hydroxycoumarin (≥98%), 4′-hydroxydiclofenac (≥98%), sulfaphenazole (≥98%), quinidine (≥98%), tranylcypromine (≥98%), chlorzoxazone (≥98%), 6-hydroxychlorzoxazone (≥98%), paclitaxel (≥98%), 6β-hydroxytestosterone (≥98%), clomethiazole (≥98%), and furafylline (≥98%) were obtained from Sigma Chemical Co. Montelukast (≥98%) was obtained from Beijing Aleznova Pharmaceutical (Beijing, China). Coumarin (≥98%), diclofenac (≥98%), dextromethorphan (≥98%), and ketoconazole (≥98%) were purchased from ICN Biomedicals. Pooled HLMs were purchased from BD Biosciences Discovery Labware. All other reagents and solvents were of analytical reagent grade.

### Assay with human liver microsomes

The effect of peucedanol on the activity of CYP isoforms was investigated in HLMs with specific probe reactions summarized in Table 1, based on previously reported studies (Zhang et al. 2007; Qi et al. 2013; Zhang et al. 2017) phenacetin *O*-deethylation for CYP1A2, coumarin 7-hydroxylation for CYP2A6, testosterone 6β-hydroxylation for CYP3A4, paclitaxel 6α-hydroxylation for CYP2C8, diclofenac 4′-hydroxylation for CYP2C9, *S*-mephenytoin 4-hydroxylation for CYP2C19, chlorzoxazone dextromethorphan *O*-demethylation for CYP2D6, and 6-hydroxylation for CYP2E1. The incubation systems with the final volume of 200 μL were composed of 100 mM potassium phosphate buffer (pH 7.4), the appropriate concentration of HLMs, a corresponding probe substrate and peucedanol (or positive inhibitor for different probe reactions) and an NADPH generating system, including 1 mM NADP^+^, 10 mM glucose-6-phosphate, 1 U/mL of glucose-6-phosphate dehydrogenase, and 4 mM MgCl_2_. All incubations were performed in triplicate, and the mean values were used.

**Table 1.** CYP450 isoforms tested, maker reactions, incubation conditions and *K_m_* used in the inhibition study.

CYPs	Marker reactions	Substrate concentration (μM)	Protein concentration (mg/mL)	Incubation time (min)	Estimated *K_m_* (μM)
1A2	Phenacetin *O*-deethylation	40	0.2	30	48
2A6	Coumarin 7-hydroxylation	1.0	0.1	10	1.5
3A4	Testosterone 6β-hydroxylation	50	0.5	10	53
2C8	Paclitaxel 6α-hydroxylation	10	0.5	30	16
2C9	Diclofenac 4′-hydroxylation	10	0.3	10	13
2C19	*S*-Mephenytoin 4-hydroxylation	100	0.2	40	105
2D6	Dextromethorphan *O*-demethylation	25	0.25	20	4.8
2E1	Chlorzoxazone 6-hydroxylation	120	0.4	30	126

Probe substrates and positive inhibitors (except for dextromethorphan and quinidine which were dissolved in water) and peucedanol (100 μM) were dissolved in methanol, with a final concentration of 1% (v/v), and 1% neat methanol was added to the incubations without inhibitor. The positive inhibitor concentrations were as follows: 10 μM furafylline for CYP1A2, 10 μM tranylcypromine for CYP2A6, 1 μM ketoconazole for CYP3A4, 5 μM montelukast for CYP2C8, 10 μM sulfaphenazole for CYP2C9, 50 μM tranylcypromine for CYP2C19, 10 μM quinidine for CYP2D6, 50 μM clomethiazole for CYP2E1. The final microsomal protein concentration and incubation time for the different probe reactions are shown in Table 1.

After preincubating for 3 min at 37 °C, a NADPH-generating system was added to start the reaction. The reaction was terminated after 30 min by adding a 100 μL acetonitrile (10% trichloroacetic acid for CYP2A6) internal standard mix, and the solution was placed on ice. Then, the mixture was centrifuged at 12,000 rpm for 10 min, and an aliquot (50 μL) of supernatant was transferred for HPLC analysis. The instrument used in this study was Agilent 1260 series instrument with DAD and FLD detector, and the quantitative assay for the corresponding metabolites was performed as previously reported (Lang et al. 2017; Zhang et al. 2017). The corresponding conditions are summarized in Table 2, and the HPLC profile results are shown in Supplementary materials.

**Table 2.** HPLC conditions for the analyses of CYP450s corresponding metabolics.

CYP450s	Conditions
1A2	methanol : phosphate buffer = 30: 70; UV 245 nm
2A6	acetonitrile : Acetic acid = 35: 65; fluo Ex/Em 340/456 nm
3A4	methanol : water = 60: 40; UV 254 nm
2C8	methanol : water = 65: 35; UV 230 nm
2C9	acetonitrile : phosphate buffer = 35: 65; UV 280 nm
2C19	methanol : potassium phosphate = 32: 68
2D6	acetonitrile : phosphate buffer = 25: 75; fluo Ex/Em 235/310 nm
2E1	acetonitrile : Acetic acid = 22: 78; UV 287 nm

### Dose-dependent study and kinetic studies of peucedanol

Peucedanol (100 μM) was incubated with eight CYP isoforms to screen CYPs activity of which was inhibited by peucedanol significantly. Then, the kinetic studies were conducted with 0, 2.5, 5, 10, 25, 50, and 100 μM peucedanol and different concentration of probe substrates (20–100 μM of phenacetin for CYP1A2, 20–100 μM of testosterone for CYP3A4, and 10–50 μM of dextromethorphan for CYP2D6) to obtain the half inhibition concentration (*IC_50_*) and *K_i_* values of the CYPs inhibited by peucedanol.

### Time-dependent inhibition study of peucedanol

A time-dependent study was performed to investigate the effect of incubation time on the inhibition by peucedanol. Briefly, 20 μM peucedanol was pre-incubated with HLMs (1 mg/mL) in the presence of an NADPH-generating system for 30 min at 37 °C. Then, an aliquot (20 μL) was transferred to another incubation tube (final volume 200 μL) containing an NADPH-generating system and probe substrates whose final concentrations were approximate to *K_m_* and continued incubation to determine the residual activity. After incubating for 0, 5, 10, 15, and 30 min, the reactions were terminated by adding a 100 μL acetonitrile internal standard mix and then placed on ice; the corresponding metabolites were determined by HPLC (Table 2).

To determine the *K_i_* and *K_inact_* values for the inactivation of CYP3A4, the incubations were conducted using higher probe substrate concentrations (approximately 4-fold *K_m_* values) and various concentrations of peucedanol (0–50 μM) after different preincubation times (0–30 min), with a two-step incubation scheme, as described above.

### Statistical analysis

The enzyme kinetic parameters for the probe reaction were estimated from the best fit line, using least-squares linear regression of the inverse substrate concentration versus the inverse velocity (Lineweaver-Burk plots), and the mean values were used to calculate *V_max_* and *K_m_*. Inhibition data from the experiments that were conducted using multiple compound concentrations were represented by Dixon plots, and inhibition constant (*K_i_*) values were calculated using non-linear regression according to the following equations:

$$v=\left(V_{\max}S\right)/\left(K_{m}\left(1\;+\;I/K_{i}\right)+S\right)\;\text{for}\;\text{competitive}\;\text{inhibition},$$

$$v=\left(V_{\max}S\right)/\left[K_{m}+S\left(1+I/K_{i}\right)\right]\;\text{for}\;\text{non}-\text{competitive}\;\text{inhibition},$$

where I is the concentration of the compound, *K_i_* is the inhibition constant, S is the concentration of the substrate, and *K_m_* is the substrate concentration at half the maximum velocity (*V_max_*) of the reaction. For the time-dependent inhibition, the *K_i_* and *K_inact_* of CYP3A4 was calculated by the following equation:

$$1/K_{\mathrm{obs}}\;=K\mathrm{\_i}/K_{\mathrm{inact}}\times 1/\left[I\right]+1/K_{\mathrm{inact}}$$

where *K_obs_* is the pseudo-first-order rate constant of inactivation at inactivated concentration [I], *K_inact_* is the maximum inactivation rate (a theoretical value that cannot be experimentally observed), and *K_i_* is the inactivated concentration when the rate of inactivation reaches half of *K_inact_*. The mechanism of the inhibition was inspected using the Lineweaver-Burk plots and the enzyme inhibition models. All experiments were performed in triplicate and the data comparison was performed using Student’s *t*-test or ANOVA and performed using IBM SPSS statistics 20 (SPSS Inc.).

## Results

### Peucedanol inhibits the activity of CYP1A2, 2D6, and 3A4

**Figure 2.**
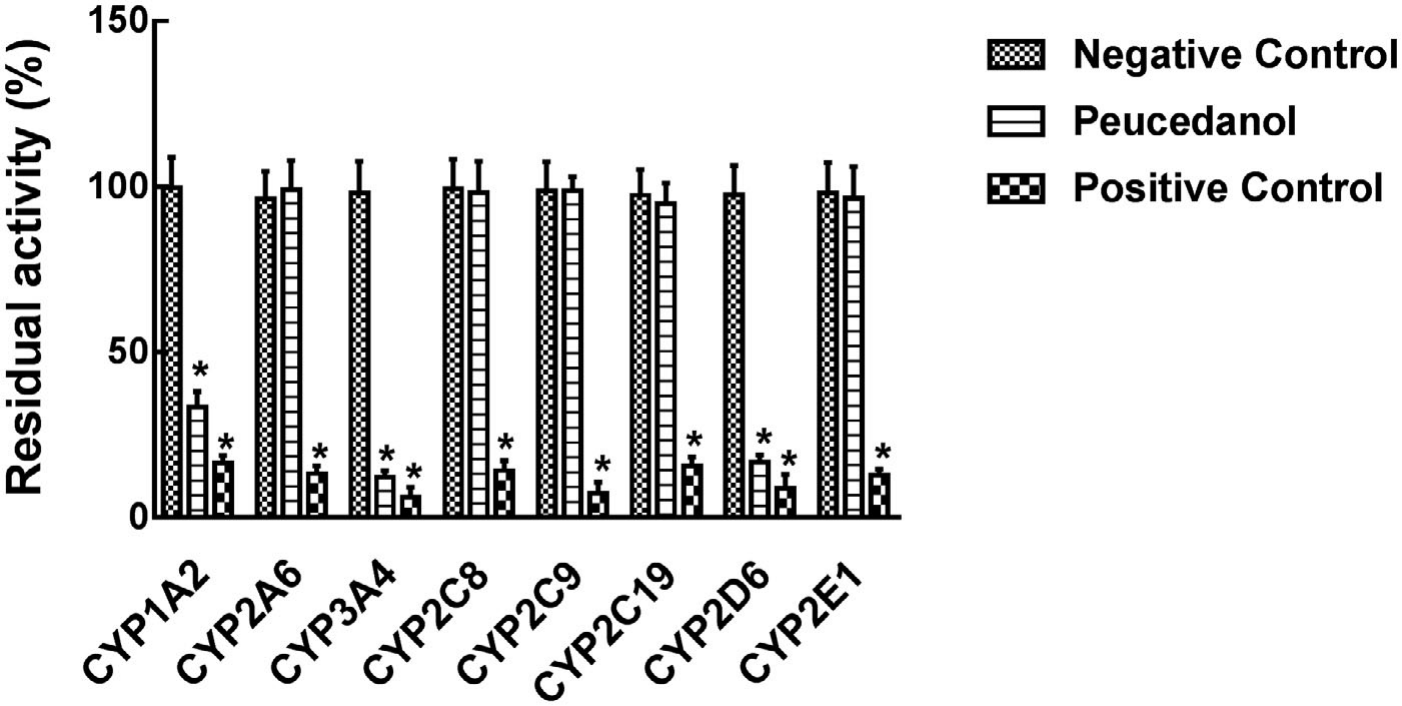
Effect of peucedanol on the activity of eight CYP isoforms, including CYP1A2, 2A6, 3A4, 2C8, 2C9, 2C19, 2D6, and 2E1. *p < 0.05. Negative control: without any treatment; Peucedanol: treated with 100 μM peucedanol; Positive control: treated with corresponding inhibitors.

**Figure 3.**
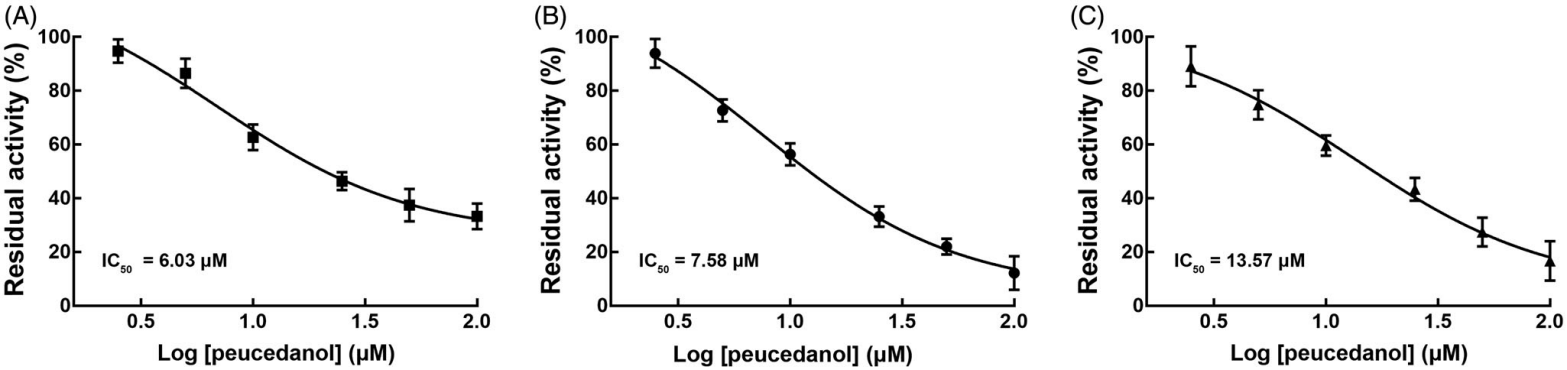
The inhibition of CYP1A2 (A), 3A4 (B), and 2D6 (C) by peucedanol was in a dose-dependent manner, with the IC_50_ values of 6.03, 7.58, and 13.57 μM, respectively.

The effect of peucedanol and specific inhibitor on the activity of CYPs was investigated in pooled HLMs. The typical inhibitors significantly decreased the activity of all CYP isoforms compared with control (*p* < 0.05). Peucedanol showed a significant inhibitory effect on the activity of CYP1A2, 2D6, and 3A4, of which the activity decreased to 33.32%, 16.73%, and 12.23%, respectively, whereas other CYPs were not affected by peucedanol (*p* < 0.05, Figure 2). Additionally, the inhibition of CYP1A2, 3A4, and 2D6 was demonstrated in a dose-dependent manner and the IC_50_ values were obtained as 6.03, 7.58, and 13.57 μM (Figure 3).

### The competitive inhibition of CYP1A2 in K_i_, and 2D6 and non-competitive inhibition of CYP3A4

**Figure 4.**
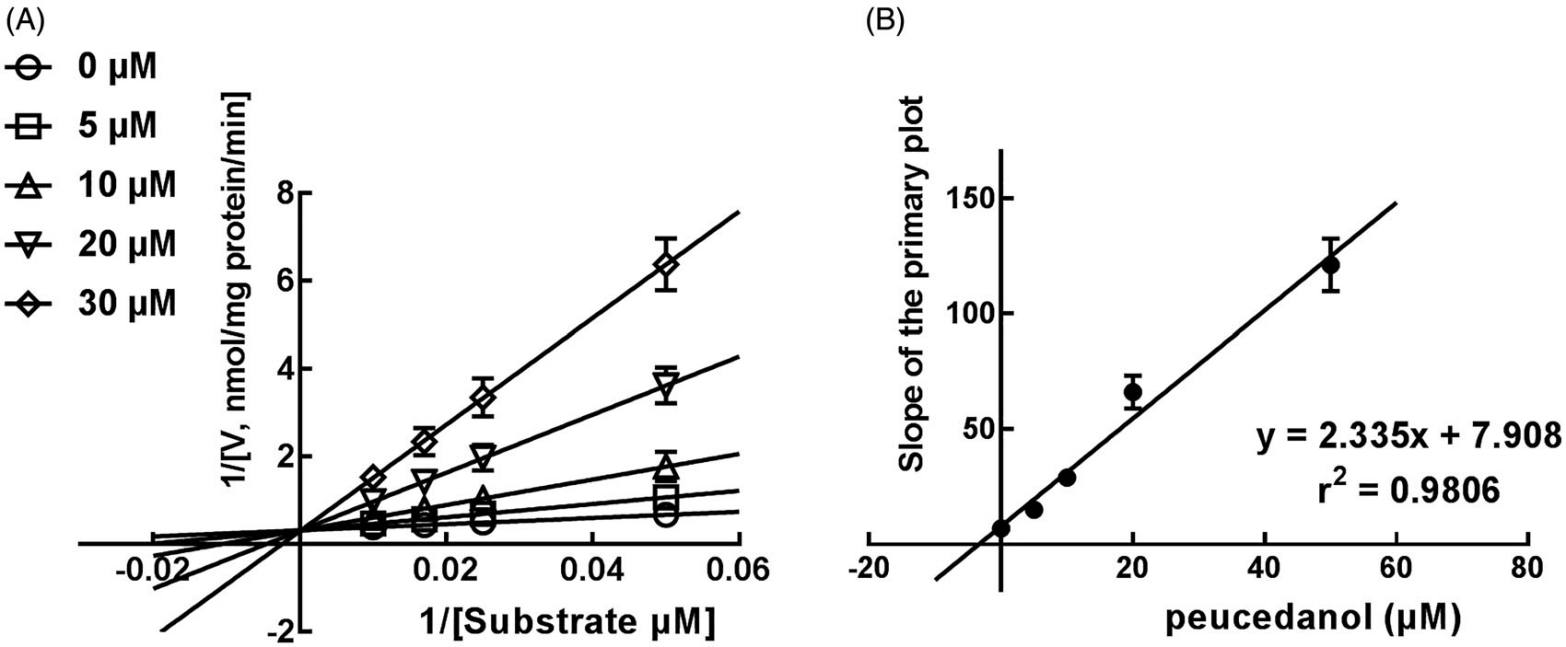
The inhibition model fitting and corresponding parameters calculation of CYP1A2 catalysed reactions (phenacetin *O*-deethylation). (A) Lineweaver-Burk plots of inhibition of peucedanol on CYP1A2 with phenacetin (20–100 μM) in the absence or presence of peucedanol (0–30 μM). The inhibition of CYP1A2 was best fitting with competitive manner. (B) Secondary plot with the values of *K_m_*/*V_max_* in Figure 4(A). All data represent the mean of the incubations (performed in triplicate).

**Figure 5.**
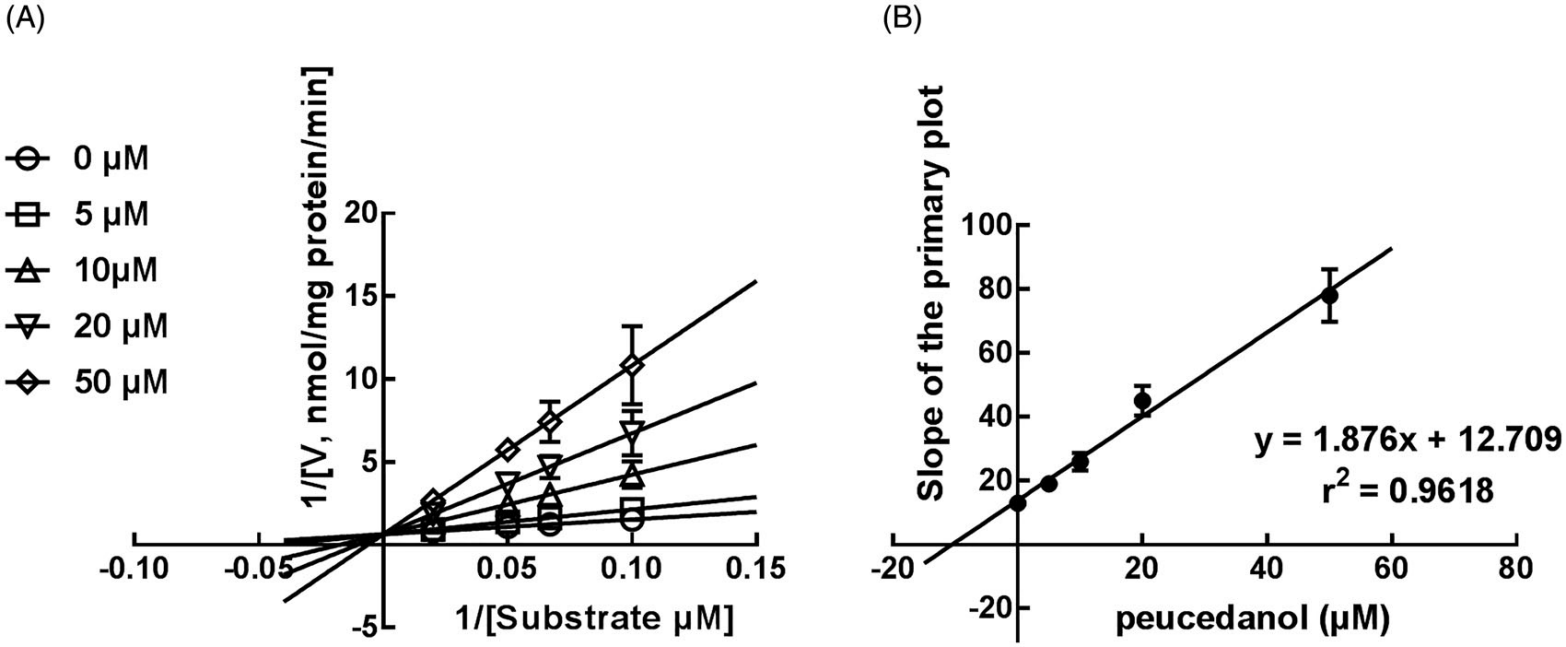
The inhibition model fitting and corresponding parameters calculation of CYP2D6 catalysed reactions (diclofenac 4′-hydroxylation) in pooled HLM. (A) Lineweaver-Burk plots of inhibition of peucedanol on CYP2D6 with dextromethorphan (10–50 μM) in the absence or presence of peucedanol (0–50 μM). The inhibition of CYP2D6 was best fitting with competitive manner. (B) Secondary plot with the values of *K_m_*/*V_max_* in Figure 5(A). All data represent the mean of the incubations (performed in triplicate).

**Figure 6.**
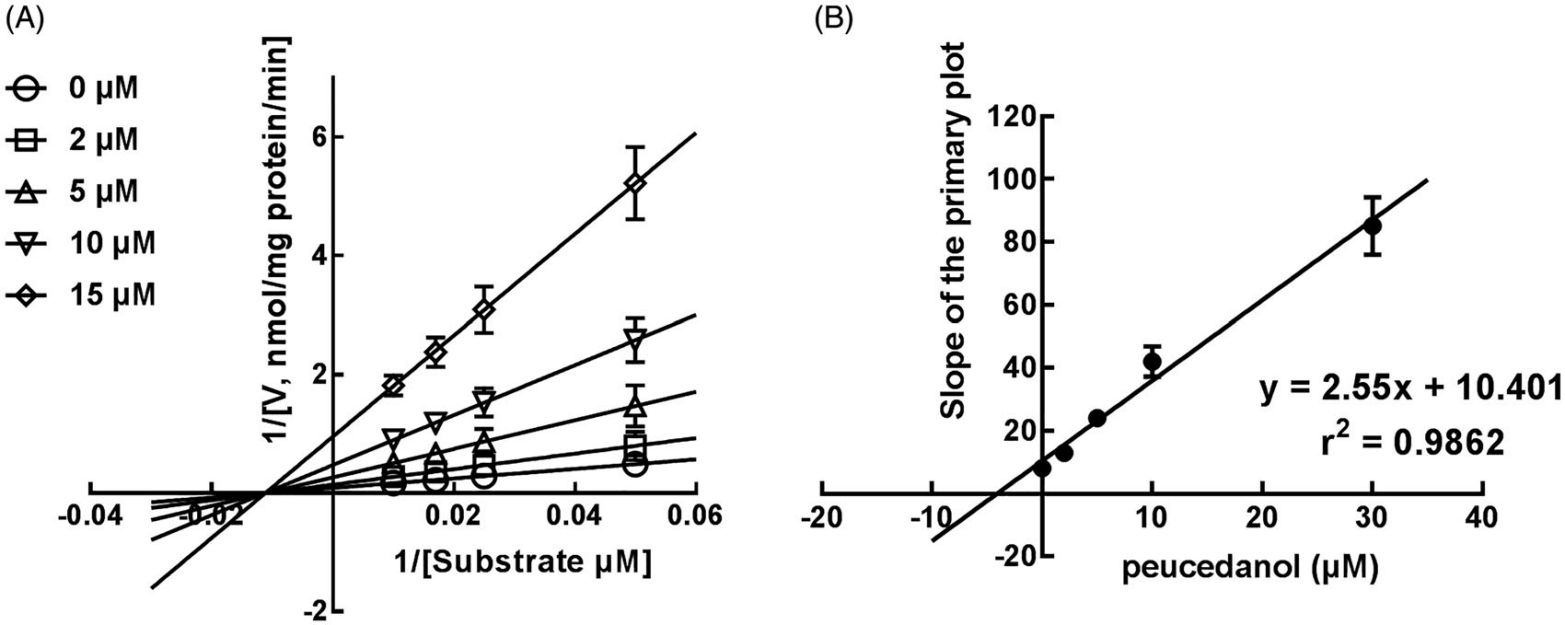
The inhibition model fitting and corresponding parameters calculation of CYP3A4 catalysed reactions (testosterone 6 b-hydroxylation) in pooled HLM. (A) Lineweaver-Burk plots of the of inhibition of peucedanol on CYP3A4 with testosterone (20–100 μM) in the absence or presence of peucedanol (0–30 μM). The inhibition of CYP3A4 was best fitting with non-competitive manner. (B) Secondary plot with the value of *K_m_*/*V_max_* in Figure 6(A). All data represent the mean of the incubations (performed in triplicate).

The Lineweaver-Burk plots of CYP1A2 and 2D6 inhibition showed that peucedanol was a competitive inhibitor of CYP1A2 and 2D6 (Figures 4(A) and 5(A)). Additionally, the *K_i_* values of CYP1A2 and 2D6 were obtained as 3.39 and 6.77 μM, respectively (Figures 4(B) and 5(B)). In contrast, the inhibition of CYP3A4 was fitted in a non-competitive manner with the *K_i_* value of 4.07 μM (Figure 6(A,B)).

### The time-dependent inhibition of CYP3A4

**Figure 7.**
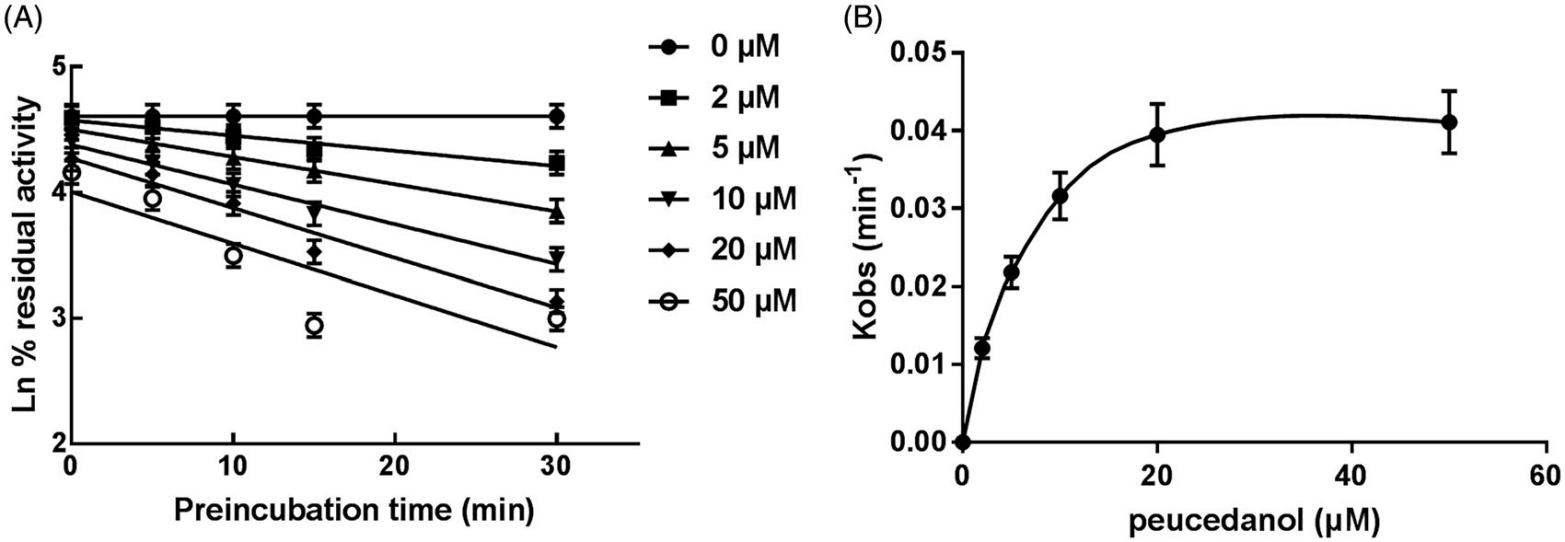
Time and concentration-inactivation of microsomal CYP3A4 activity by peucedanol in the presence of NADPH. (A) The initial rate constant of inactivation of CYP3A4 by each concentration (*K_obs_*) was determined through linear regression analysis of the natural logarithm of the percentage of remaining activity versus pre-incubation time. (B) The *K_i_* and *K_inact_* values were determined through non-linear analysis of the *K_obs_* versus the peucedanol concentration.

CYP1A2, 2D6, and 3A4 were incubated with peucedanol for different time. It was found that the activity of CYP3A4 significantly decreased with the incubation time, whereas CYP1A2 and 2D6 were not affected indicating the inhibition of CYP3A4 was time-dependent. Moreover, the corresponding parameters of the time-dependent inhibition of CYP3A4 were obtained by the non-linear regression analysis (Figure 7(A,B)). The value of *K_i_*/*K_inact_* of CYP3A4 was 5.44/0.046 min/μM means approximately 4.6% CYP3A4 was inactivated each minute when incubating with a saturating concentration of peucedanol in HLMs.

## Discussion

In Chinese traditional medicine, the concomitant use of herbal medicines may induce clinically relevant herb-drug interaction or adverse reaction or even metabolic disorders, when some herbs are co-administrated with those drugs with narrow therapeutic indices (Chua et al. 2015; Ma and Ma 2016; Li et al. 2019). Therefore, because of the centrally important role of CYPs in drug-drug interaction and toxicology studies, it is necessary to assess the CYPs inhibition or induction potential of commonly used drugs or herbs.

CYP1A2, 2D6, and 3A4 are major isoforms of CYP involved in the metabolism of a large number of drugs, such as mexiletine, tamoxifen, and amlodipine (Nakajima et al. 1998; Zhu et al. 2014; MacLeod et al. 2017). Previously, CYP3A4 has been reported to mediate the drug-drug interaction between verapamil and oridonin result in the increase of oridonin exposure (Liu et al. 2019). Peucedanol was found to be a non-competitive inhibitor of CYP3A4 in this study and the inhibitory effect of peucedanol was demonstrated to be time-dependent indicating the incubation time is an important factor in the interaction between peucedanol and CYP3A4. CYP3A4 metabolizes a large variety of paediatric drugs, which makes it possible that peucedanol may co-administrated with CYP3A4 substrates (Rafaniello et al. 2018; Faria et al. 2019).

In addition, peucedanol also exerted a significant inhibitory effect on the activity of CYP1A2 and 2D6 in a competitive manner. The different inhibitory manners of peucedanol in CYP1A2, 2D6, and 3A4 may be a result of the chemical structure of peucedanol. Although these two CYP isoforms correspond to a minor part of CYPs, they are also involved in the metabolism of a variety of drugs, which significantly increase the potential for drug-drug interaction (Urichuk et al. 2008).

Besides the CYPs in the liver, the intestinal CYPs also play vital roles in the metabolism of drugs (Thelen and Dressman 2009). These studies investigated the interaction between peucedanol and CYP enzymes in pooled HLMs, the effect of peucedanol on the activity of intestinal CYPs should be paid attention in further study, which can provide more clinical guidance for the use of peucedanol. Moreover, the effect of peucedanol on the activity of CYPs was studied *in vitro* in this research. Further *in vivo* studies are needed to assess the risk of potential interaction and verify the *in vitro* results obtained in this study.

## Conclusions

The significant inhibitory effect of peucedanol on the activity of CYP1A2, 2D6, and 3A4 was found in the present study. The inhibition of CYP1A2, 2D6, and 3A4 was found to perform in a dose-dependent manner with various IC_50_ values. Moreover, the inhibition of CYP3A4 was demonstrated to be time-dependent, which indicated that the incubation time is an important factor in the interaction between peucedanol and CYP3A4. These results implied the potential herb-drug or herb-herb interaction might occur during the co-administration between peucedanol and other drugs metabolized by CYP1A2, 2D6, or 3A4, which needs further *in vivo* validation.

## Supplementary Material

SI_Figure_8.tif

SI_Figure_7.tif

SI_Figure_6.tif

SI_Figure_5.tif

SI_Figure_4.tif

SI_Figure_3.tif

SI_Figure_2.tif

SI_Figure_1.tif
