# Supplementary figures and images for: In vitro study on the effect of peucedanol on the activity of cytochrome P450 enzymes
Source: Pharm Biol. 2021 Jul 10;59(1):935–40. doi: 10.1080/13880209.2021.1944223 (PMC8274509; doi:10.1080/13880209.2021.1944223)

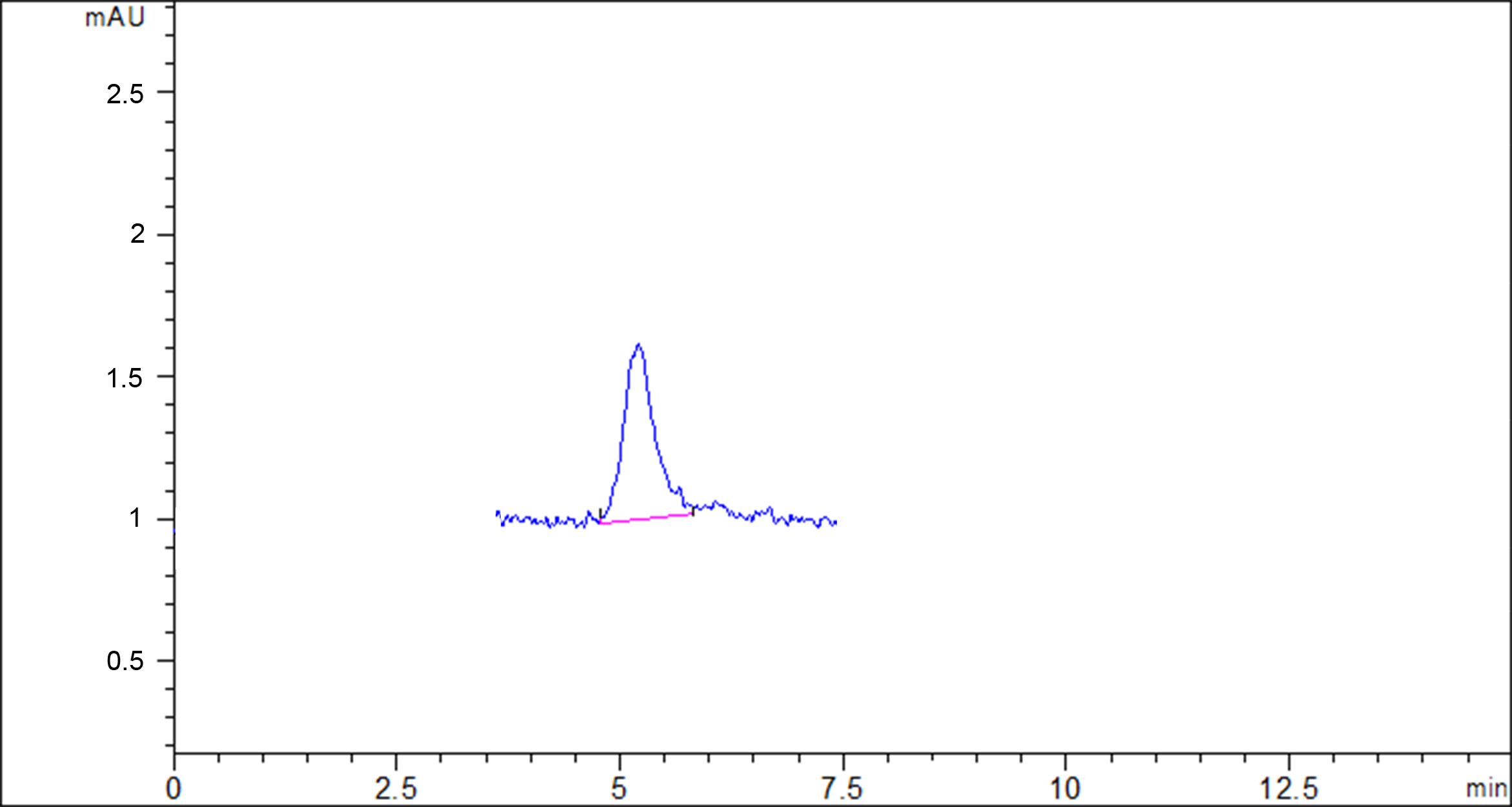

Supplement: SI_Figure_8.tif [file IPHB_A_1944223_SM9486.tif]

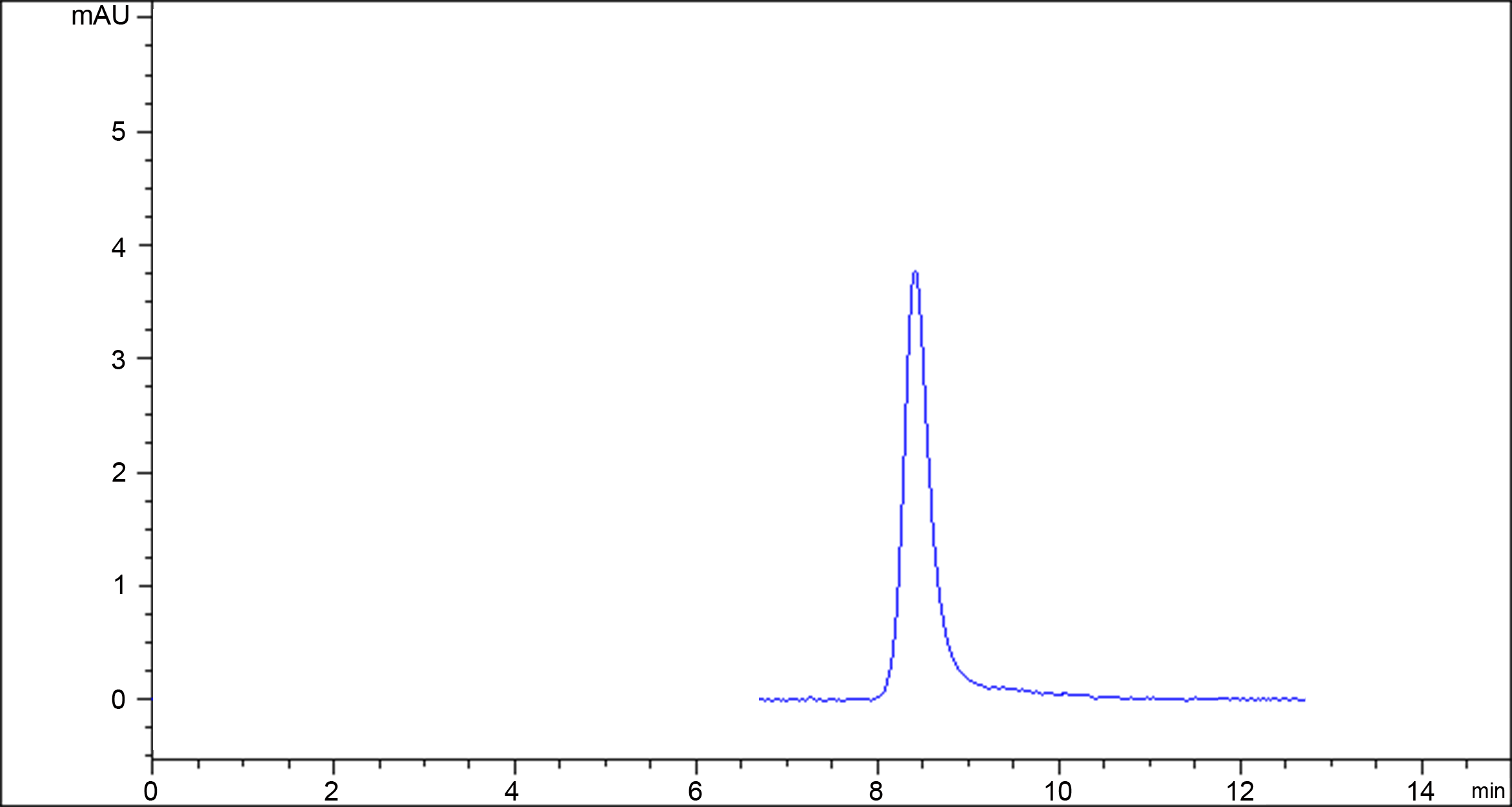

Supplement: SI_Figure_7.tif [file IPHB_A_1944223_SM9485.tif]

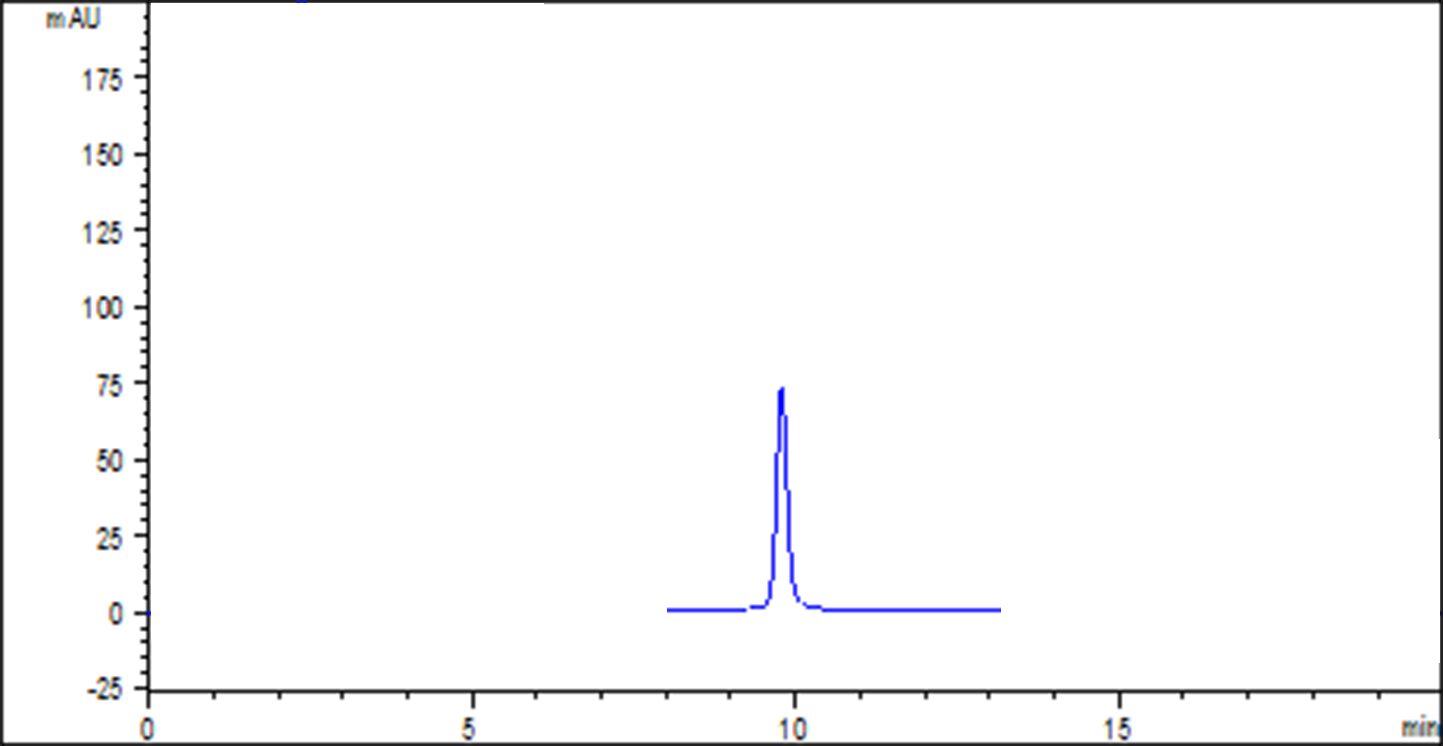

Supplement: SI_Figure_6.tif [file IPHB_A_1944223_SM9484.tif]

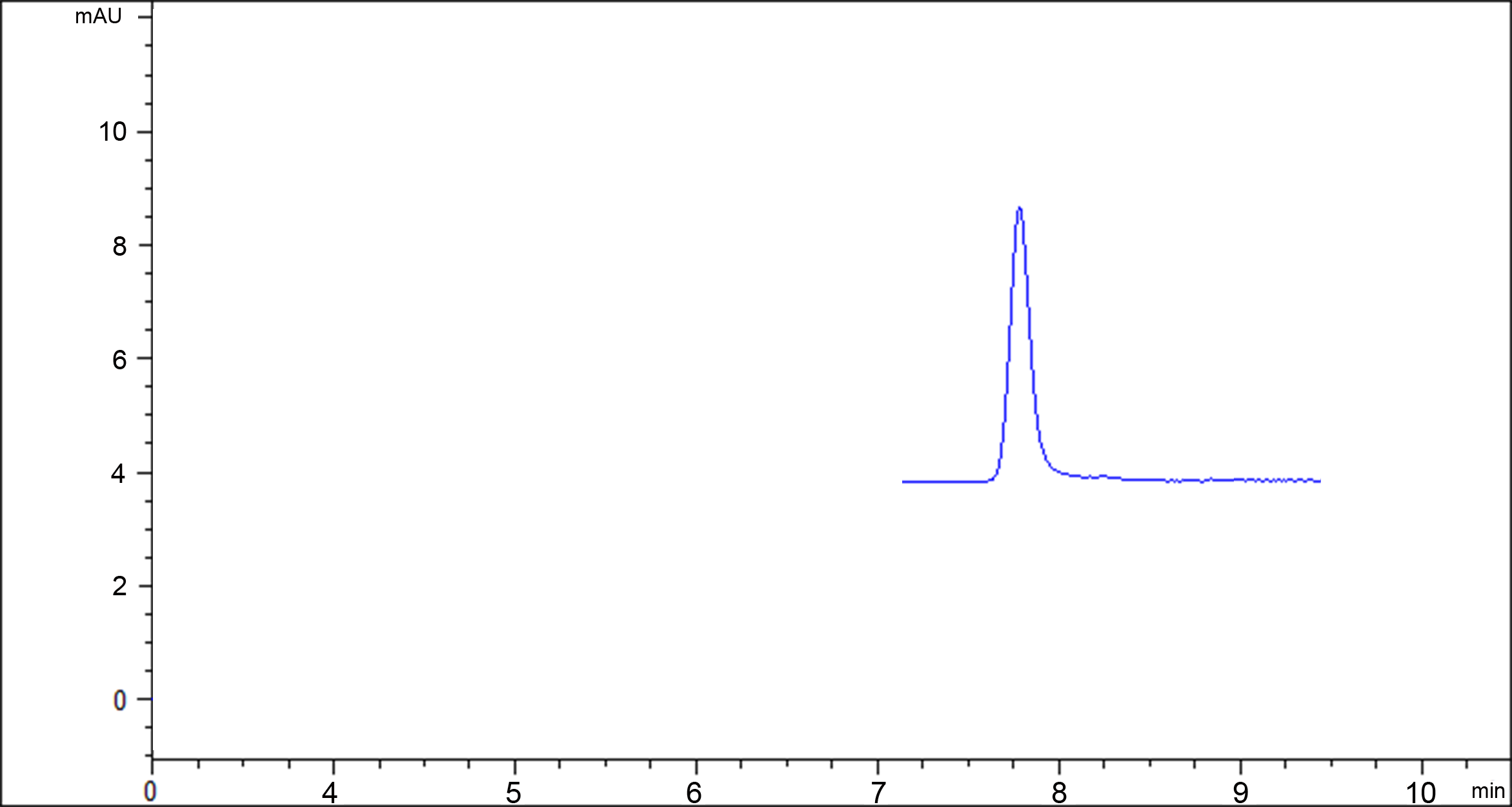

Supplement: SI_Figure_5.tif [file IPHB_A_1944223_SM9483.tif]

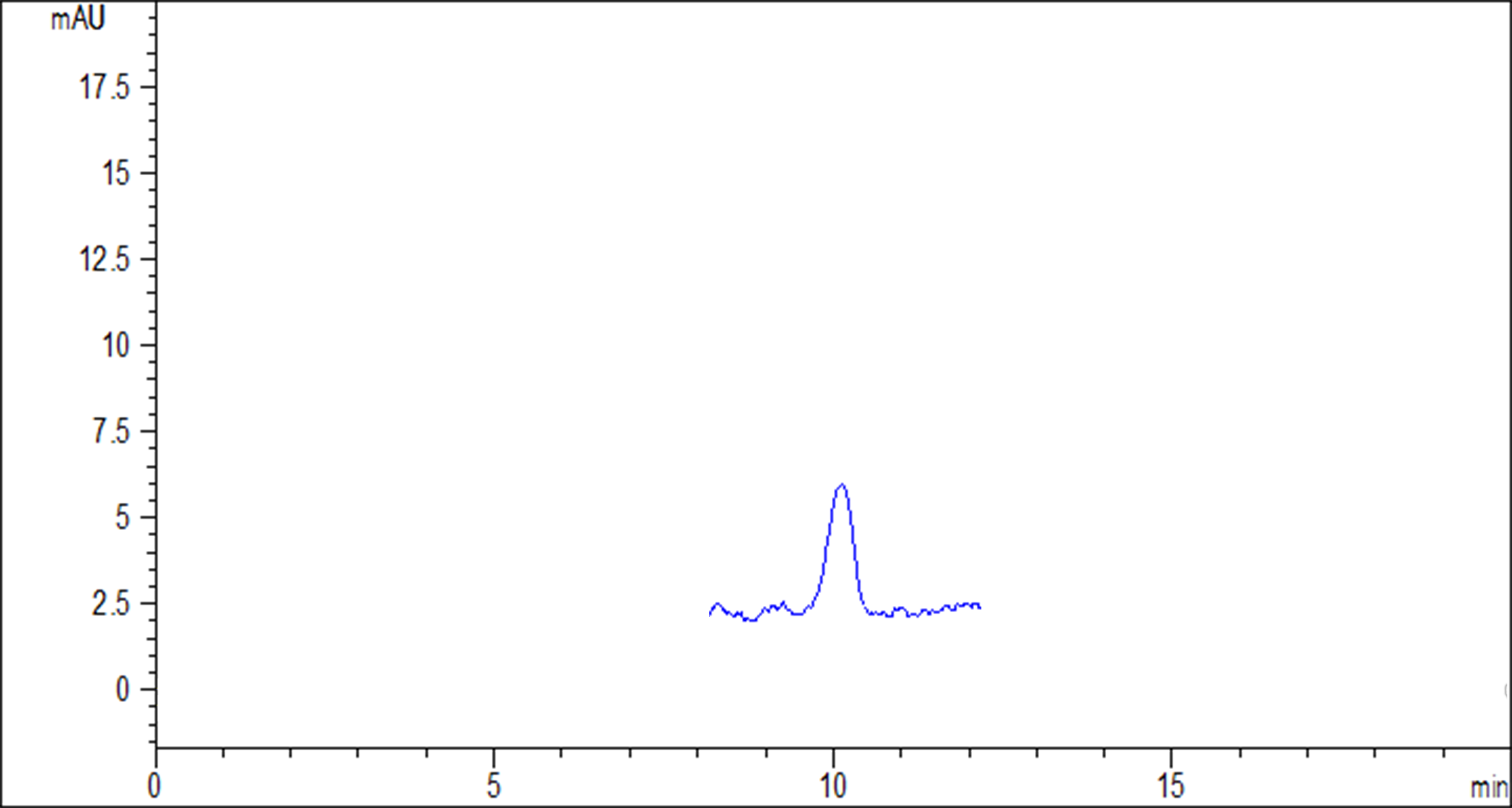

Supplement: SI_Figure_4.tif [file IPHB_A_1944223_SM9482.tif]

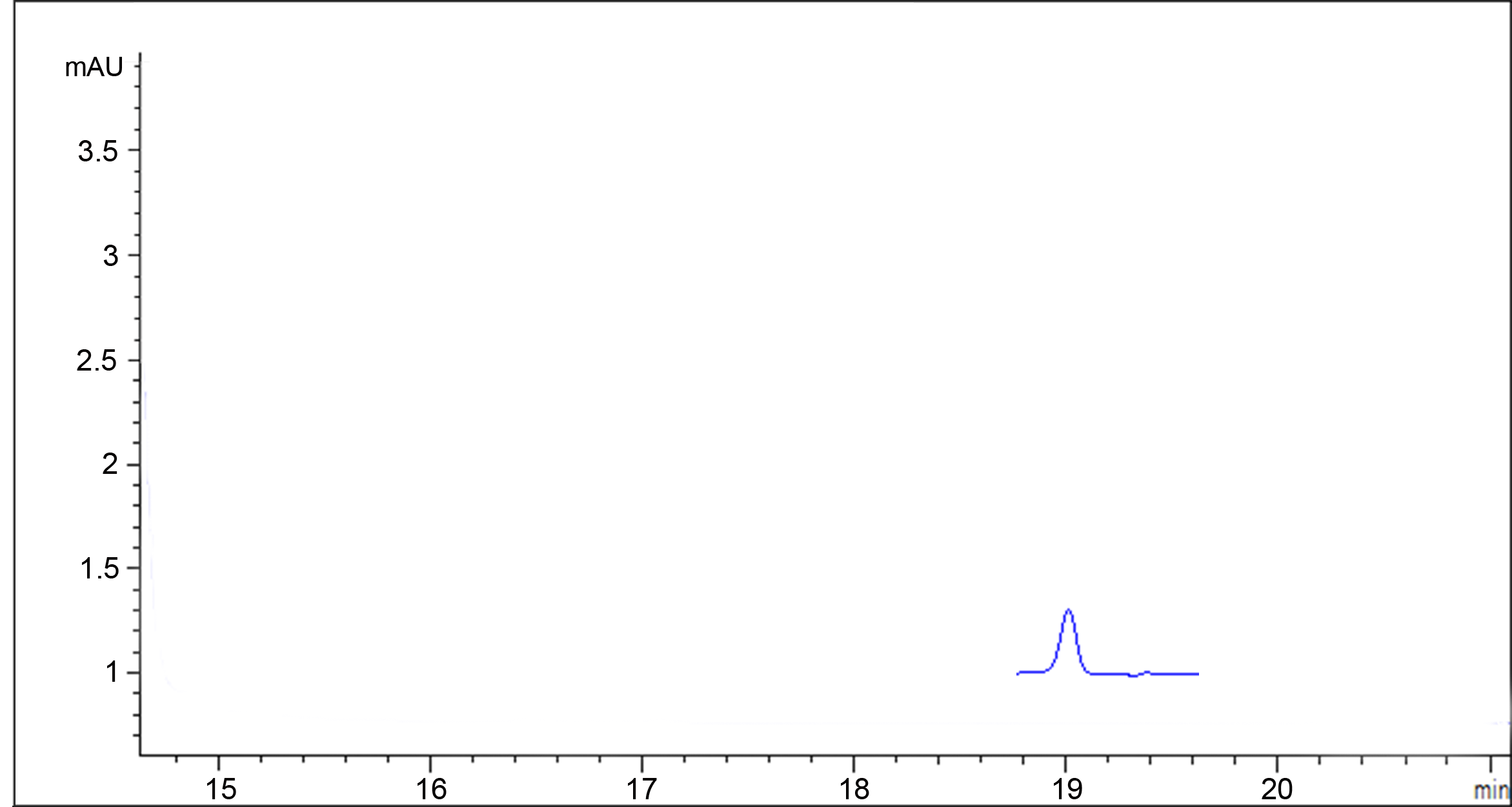

Supplement: SI_Figure_3.tif [file IPHB_A_1944223_SM9481.tif]

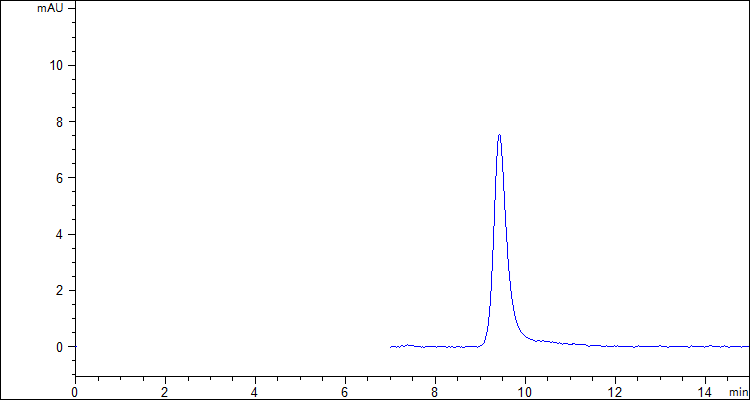

Supplement: SI_Figure_2.tif [file IPHB_A_1944223_SM9480.tif]

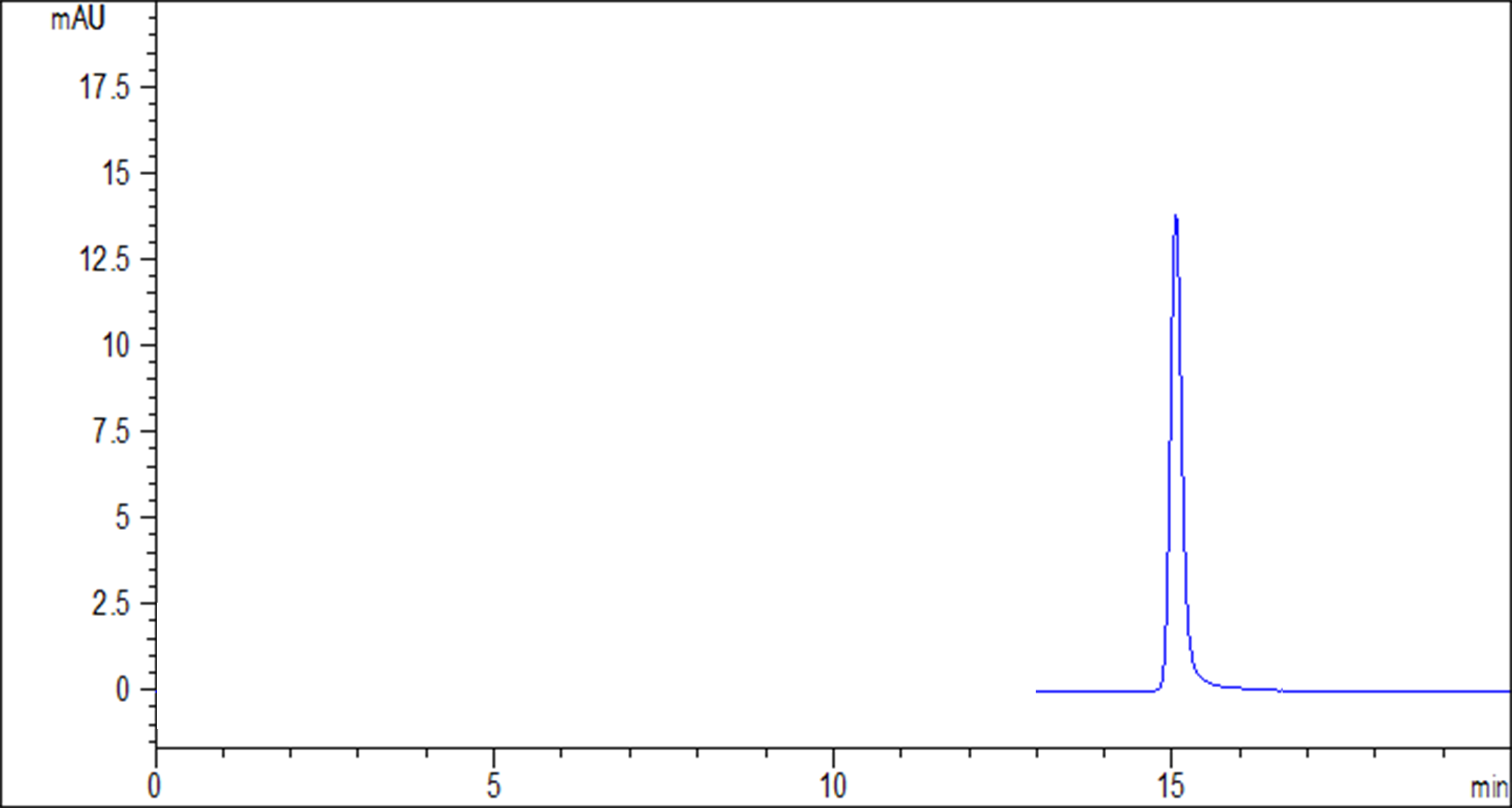

Supplement: SI_Figure_1.tif [file IPHB_A_1944223_SM9479.tif]
